# Reliability of AI Methods in Drug Discovery: Evaluation of Boltz‑2 for Structure and Binding Affinity Prediction

**DOI:** 10.1021/acs.jctc.6c01334

**Published:** 2026-07-29

**Authors:** Shunzhou Wan, Xibei Zhang, Xiao Xue, Peter V. Coveney

**Affiliations:** † Centre for Computational Science, 4919 University College London, London, WC1H 0AJ, United Kingdom; ‡ Advanced Research Computing Centre, University College London, London, WC1H 0AJ, United Kingdom

## Abstract

Despite continuing hype about the role of AI in drug discovery, no “AI-discovered drugs” have so far received regulatory approval. Here we assess one of the latest AI-based tools in this domain. Boltz-2, a recently developed biomolecular foundation model, aims to bridge the gap between AI efficiency and physics-based precision through a joint “cofolding” approach. In this study, we provide an extensive evaluation of Boltz-2 using two large-scale data sets: 16780 compounds for 3CLPro and 21702 compounds for TNKS2. We compare Boltz-2 predicted structures with traditional docking and binding affinities with binding free energies derived from the physics-based ESMACS protocol. Structural analysis reveals significant global RMSD variations, indicating that Boltz-2 predicts multiple protein conformations and ligand binding positions rather than a single converged pose. Energetic evaluations exhibit only weak to moderate correlations across the global data sets. Furthermore, a focused analysis of the top 100 compounds yields no significant correlation between the Boltz-2 predictions and the binding free energies from fine-grained ESMACS, alongside frequently observed saturation-state errors in Boltz-2 predicted ligand structures. Our results show that Boltz-2 lacks the energetic resolution required for lead identification. These findings highlight the necessity of employing physics-based methods for the reliability and refinement of AI-derived models.

## Graphical abstract

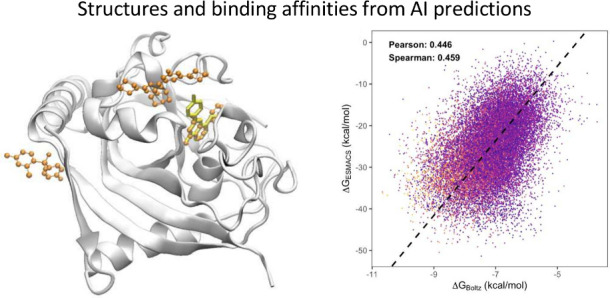

## 1 Introduction

More than a decade has passed since the initial wave of optimistic projections suggested that artificial intelligence (AI) would fundamentally transform the landscape of drug discovery.^1,2^ During this period, a wide range of promising tools and deep-learning architectures have proliferated, claiming the ability to revolutionize every stage of the early drug development pipeline – from the identification of novel drug targets and the prediction of their 3D structures to the rapid identification and optimization of lead compounds.^3^ These efforts reached a significant milestone with the development of AlphaFold 2,^4^ a breakthrough in the high-fidelity prediction of protein structures that was subsequently recognized with the 2024 Nobel Prize in Chemistry.^5^ However, despite such unprecedented successes, the impact of AI on drug discovery remains a subject of critical debate. While AlphaFold 2 provides valuable structural hypotheses that can significantly accelerate research, recent evaluations emphasize that its predictions do not replace the necessity of experimental structure determination.^6^ Consequently, the expected revolution in the delivery of novel, clinically successful therapeutics has yet to fully materialize.^7,8^ Many AI-driven predictions have failed to translate into reliable experimental outcomes, highlighting the persistent challenge of accurately quantifying the interaction energetics required for hit identification and lead optimization.^9^

Indeed, the precise determination of the binding affinities of small molecules for their target proteins remains a cornerstone of structure-based drug design. This capability directly dictates the efficiency of the entire discovery pipeline, as the precision of these energetic estimations is essential for the effective identification and subsequent optimization of novel drug candidates.^10^ Traditionally, the field has relied on a trade-off between high-throughput, lower-precision methods such as molecular docking^11^ and computationally demanding, high-precision physics-based approaches. The latter approaches include linear interaction energy (LIE),^12^ molecular mechanics energies combined with Poisson–Boltzmann or generalized Born and surface area continuum solvation (MMPBSA and MMGBSA) methods,^13^ and alchemical methods,^14^ which have been broadly applied to model molecular recognition for drug discovery and lead optimization (an example of the application of alchemical methods can be found in the literature^15^).

Standard MMPBSA or alchemical free energy calculations typically rely on one-off simulations, which can be highly sensitive to initial conditions and fail to sample the conformational landscape adequately, leading to significant statistical uncertainties and poor reproducibility.^16,17^ To bridge the gap between theoretical precision and practical reliability, we have developed ensemble-based protocols such as ESMACS (enhanced sampling of molecular dynamics with approximation of continuum solvent)^18,19^ and TIES (thermodynamic integration with enhanced sampling).^19,20^ These methods build upon the foundations of MMPBSA and thermodynamic integration by incorporating an ensemble approach to rigorously address the inherent stochastic properties of molecular dynamics (MD) simulations.^16^ By executing multiple independent replicas with varying initial configurations, ESMACS and TIES, which identify lead molecules and perform lead optimization, respectively, provide reliable and reproducible binding free energy estimates with quantification of statistical uncertainties. ESMACS has been applied to data sets of tens of thousands of compounds on exascale computers for drug discovery.^21^ Although it could be increased 10-fold without trouble, the set of compounds is still very small, compared to the vast chemical space that needs to be explored (estimated to be about 10^68^ compounds). The high computational cost of physics-based approaches limits their applications to relatively small sets of compounds, necessitating faster alternatives for large-scale screening.

Recent advances in generative artificial intelligence (AI) have substantially expanded the accessible chemical space for drug discovery,^22^ enabling the rapid design of novel compounds through deep generative models^23,24^ and reinforcement learning-based optimization.^21,25^ The development of surrogate docking approaches^26^ makes it practical to rapidly screen a vast number of compounds from existing libraries. In practice, these approaches remain critically dependent on fast surrogate scoring functions, which often lack explicit physical grounding and are liable to introduce systematic biases during optimization.^27^

While these task-specific models often function in an isolated context, there is a growing requirement for more integrated, multimodal systems that unify diverse biomolecular predictions. The emergence of “foundation models”^28^ has subsequently shifted the landscape of AI. They are large-scale architectures trained on massive, diverse data sets that can be adapted to a wide range of downstream tasks. These models are intended to serve as a versatile “foundation” for many different applications. It should be noted that, like all AI methods, foundation models cannot magically create or predict arbitrary molecules and molecular structures; they are only as good as the data they are trained on.

Building on the requirement for both precision and speed, Boltz-2 ^29^ has recently emerged as a pioneering structural biology foundation model designed to bridge the gap between AI and rigorous physics. It is categorized as a billion parameter foundation model because it is trained on a vast data set of protein structures and millions of experimental binding records. While other “cofolding” frameworks such as AlphaFold 3,^30^ RoseTTAFold All-Atom^31^ and the initial Boltz-1 release^32^ primarily focus on high-fidelity structural assembly, the unified architecture of Boltz-2 extends these capabilities to include direct, quantitative binding affinity prediction. Notwithstanding the vast array of ML methods that have been specifically designed for this purpose in recent years, achieving high-fidelity accuracy remains a significant challenge.^33^ A key strength of Boltz-2 is its claimed proficiency in ranking analogues within a chemical series, allowing it to distinguish subtle variations in potency for hit-to-lead and lead optimization. Consequently, Boltz-2 represents a potential paradigm shift in lead identification, offering a high-throughput alternative that maintains the structural and energetic nuance required for drug discovery. It is this specific claim of predictive accuracy, purportedly reaching the accuracy levels of free energy perturbation (FEP) while operating orders of magnitude faster,^29^ within a single integrated framework, that motivates the study reported here.

Since its release, Boltz-2 has attracted much attention in peer-reviewed articles,^34,35^ preprints,^36,37^ and blogs,^38,39^ which seek to evaluate the reliability of its predictions and its domains of applicability. In particular, Bret et al.^34^ demonstrated that, although Boltz-2 excels as a binary classifier, it exhibits limited precision when quantitatively ranking compounds by affinity. However, the performance of Boltz-2 across large-scale, target-specific chemical libraries remains to be rigorously validated against established physics-based benchmarks. Furthermore, drug discovery frequently explores complex molecular structures and millions of novel candidates with little or no training data, posing a substantial challenge, even for foundation models that perform well on more common molecules.

In this study, we present a comprehensive evaluation of Boltz-2’s predictive capabilities using two distinct and therapeutically relevant protein targets: the SARS-CoV-2 main protease (3CLPro), a critical enzyme for viral replication, and tankyrase 2 (TNKS2), a key regulator in Wnt (a portmanteau of “Wg” and “int” standing for “Wingless-related integration site”) signaling and a major cancer drug target. Utilizing data sets of 16780 and 21702 compounds^21^ respectively, we assess the model’s structural fidelity via root-mean-square deviation (RMSD) and local distance difference test (LDDT) comparisons against traditional docking, while simultaneously benchmarking its affinity predictions against binding free energies derived from the ESMACS protocol. The ensemble strategy of ESMACS effectively mitigates the sensitivity of single-trajectory simulations to initial conditions and enables UQ assessment which is entirely absent in standard AI methods. It therefore provides a robust benchmark for assessing the performance of the AI-driven affinity predictors. By examining both global correlations and the precision of top-ranked candidates, we aim to establish whether Boltz-2 can serve as a reliable, high-speed surrogate for physics-based pipelines in virtual screening phase of drug discovery.

## 2 Methods

We describe in this section the data sets of compounds used for the benchmarking of the Boltz-2 foundation model against established physics-based methodologies, the execution of the ensemble-based ESMACS protocol for affinity free energy calculations, and the specific metrics employed to quantify the structural and energetic divergence between AI-driven and traditional approaches.

### 2.1 Data Set Preparation

The data sets for 3CLPro and TNKS2 were obtained from a large-scale compound library previously described by Loeffler et al.^21^ While full details are available in the original study, the generation process is summarized as follows.

The two data sets consist of initially ∼10000 compounds each, screened from ZINC15 and MCULE compound databases using a surrogate docking model^40^ for 3CLPro or generated using REINVENT,^41^ a generative molecular AI, based on 27 known compounds with available experimental affinity measurements for TNKS2. More compounds were then generated iteratively with REINVENT, which uses reinforcement learning (RL), to optimize molecules subjected to external “information”, i.e., scoring functions that evaluate each compound for its fitness. The scoring function used was the predicted binding affinities from ESMACS simulations (see section 2.3). In each generative active learning (GAL) iteration, a new batch of compounds was produced with improved fitness. Using various batch sizes in each GAL cycle and the number of cycles to reach convergent behavior toward the end of GAL, approximately 16900 and 21861 compounds were studied in total for 3CLPro and TNKS2, respectively. We removed the compounds that encountered execution errors during the molecular dynamics simulation or the free energy calculation steps, resulting in final data sets of 16780 and 21702 compounds for the two molecular systems (∼0.7% failures).

To rigorously evaluate the reliability and predictive accuracy of both the ESMACS and Boltz-2 protocols against true physical baselines, independent experimental validation data sets were also compiled. Target-specific compounds with known experimental measurements were extracted from the BindingDB database, which contains aggregated data from other highly public repositories like ChEMBL and PubChem. This retrieval yielded 59 compounds for 3CLPro and 1137 compounds for TNKS2 (as of June 1, 2026), which were subsequently utilized to benchmark the correlation between experimental values and our computational predictions. It should be noted that the compounds deposited before June 2023 had been used for Boltz-2 training. Consequently, these two subsets must be evaluated carefully to distinguish true biophysical generalizability from potential machine-learning memory. To account for this, the data points originating before and after this training cutoff date are treated separately and distinguished by different colors in our subsequent evaluation plots.

### 2.2 Boltz-2 Inference and Structure Prediction

Predictive modeling and binding affinity prediction were conducted using Boltz-2. For each ligand in the 3CLPro and TNKS2 data sets, the model was provided with the protein sequence and SMILES strings for the ligands. Unlike traditional docking, Boltz-2 utilizes a cofolding architecture that allows for reciprocal conformational changes in both the protein and the ligand during the prediction process.

Inference was performed using the default pretrained model weights, with no spatial constraints applied, to evaluate its “out-of-the-box” performance on large-scale screening tasks. For each compound, we extracted the predicted binding affinity (reported as a continuous score) and the 3D atomic coordinates of the pose with the highest confidence. Predicted binding affinities, reported as log_10_(IC_50_) values with IC_50_ in micromolar units (μM), can be converted to pIC50 in kcal/mol using formula (6 – log_10_(IC_50_)) × 1.364.

Boltz-2 predictions were performed on Isambard-AI, which is made up of 5448 NVIDIA GH200 Grace-Hopper superchips at the University of Bristol. In order to improve computational efficiency, UniProt Reference Clusters at 90% sequence identity (UniRef90)^42^ were used to generate local multiple sequence alignments. UniRef90 is a clustered protein database from UniProt, where sequences with no less than 90% identity are merged to reduce redundancy while retaining representative diversity for the known protein sequence space. Boltz-2 inference required approximately 77 s per compound for 3CLPro and 74 s for TNKS2 to predict protein–ligand structures and binding affinities on a single GH200 GPU. To accelerate the screening process, we used 32 GPUs concurrently, providing a proportionate reduction in the total wall-clock time for the full data set.

### 2.3 ESMACS Calculations

The initial ESMACS binding free energies were extracted from our previous molecular design study,^21^ in which a coarse-grained ESMACS protocol was employed to optimize computational efficiency. The standard ESMACS protocol^18^ uses an ensemble of 25 replicas and 4 ns production run for each compound-protein complex to get precise predictions. For a drug screening project, like our previous study,^21^ tens of thousands of compounds need to be evaluated. A coarse-grained protocol (CG-ESMACS), using an ensemble of 10 replicas and smaller buffer distance of 10 Å, is deployed, which inevitably decreases the precision of the predictions but is well-suited for high-throughput virtual screening. Note that the time-consuming entropic calculation is omitted in the current ESMACS protocol as its inclusion, although it brings the calculated binding free energies closer to their experimental measurements, does not improve their rankings in general.^43,44^

To further investigate the structural and energetic precision of Boltz-2 in a hit-to-lead context, a more rigorous, fine-grained ESMACS (FG-ESMACS) protocol was applied to a selected subset consisting of the top 100 compounds from the Boltz-2 predictions for each target. This focused evaluation employed a robust ensemble approach to minimize statistical uncertainty and provide a high-resolution validation. For each of the 200 selected protein–ligand complexes, an ensemble of 25 replicas was used in FG-ESMACS. A 10 ns production run was performed for each replica, totaling 250 ns of sampling per compound. Three sets of simulations were performed using distinct initial structures: (1) those generated via docking, (2) Boltz-2 structures with directly added hydrogen atoms, and (3) Boltz-2 structures with hydrogen placements constrained by their SMILES strings (section 3.2.1).

For the preparation of the docking structures, the selected compounds were first processed by FixpKa^45^ to obtain the correct protonation state at pH 7.4. Subsequently, up to 200 conformers per compound were generated using OMEGA.^45^ The prepared structural library of the compounds was then docked to the protein (PDB IDs: 6W63 for 3CLPro and 4UI5 for TNKS2) using FRED.^45^ The docking poses with the best Chemgauss4 docking scores were used for ensuing ESMACS simulations. For the structures directly from Boltz-2 prediction, the assigncharges.py script from OpenEye Scientific was used to assign proper protonation states and add explicit hydrogen atoms to represent the charge distribution. To generate Boltz-2 structures with configurations derived from SMILES strings, an in-house Python script was employed to assign hydrogen atoms based on explicit chemical rules rather than on simple geometric proximity.

MD simulations were conducted using the NAMD^46^ engine and the Amber force field.^47^ The final binding free energies were calculated using AmberTools,^48^ with results averaged across the 25 replicas to ensure highly converged affinity estimates, providing a reproducible and precise benchmark for evaluating Boltz-2’s predictive accuracy. All MD simulations and free energy calculations were performed on Frontier, the world’s first exascale machine at the Oak Ridge National Laboratory in Oak Ridge, Tennessee Ridge, Tennessee, U.S.A. Frontier features 37888 AMD Instinct MI250X with each MI250X containing 2 GPUs. Our FG-ESMACS simulations, comprising 5000 individual runs, were executed concurrently across 5000 GPUs (one per run). The entire set of ensemble runs was completed within 112 and 101 min for 3CLPro and TNKS2, respectively.

### 2.4 Structural and Energetic Evaluation Metrics

To assess the predictive accuracy of Boltz-2, we employed comparative metrics and statistical analysis focusing on both the geometric fidelity of the predicted complexes and the statistical reliability of the affinity estimations. The structural divergence between the structures predicted by Boltz-2 and those obtained via traditional docking was quantified using two complementary metrics: pairwise root-mean-square deviations (RMSDs) which were calculated for the main chain of proteins and heavy atoms of ligands, following a rigid-body superposition of the protein backbone to measure global pose displacement; and local distance difference test (LDDT) which is used to assess the preservation of the chemical environment without the bias of global superposition. The LDDT metric evaluates the consistency of interatomic distances between the ligands and binding site residues within a 15 Å radius, providing a high-resolution measure of local interaction fidelity.

The correspondence between Boltz-2 predicted affinities and the binding free energies from ESMACS calculations was evaluated using two primary statistical indicators: the Pearson correlation coefficient (*r*) which measures the strength of the linear relationship between the Boltz-2 predictions and the physics-based free energies, and the Spearman’s rank correlation (ρ) which determines the model’s ranking fidelity, assessing its ability to correctly order compounds by potency, a critical factor for virtual screening effectiveness. For the top-100 high-precision subset, we also compared the detailed binding poses and the binding free energies from different initial structures.

## 3 Results and Discussion

In this section, we present a quantitative assessment of Boltz-2’s performance across the two distinct protein targets: 3CLPro and TNKS2. Our evaluation focuses on two critical dimensions of the model’s output: the geometric fidelity of the predicted protein–ligand complexes and the accuracy of the estimated binding free energies. To establish a rigorous benchmark, we first perform a global analysis across the entire data set of 38482 compounds, comparing Boltz-2’s structural poses with traditional docking results and its affinity predictions with ESMACS energies. This is followed by a targeted analysis of the top 100 compounds identified by Boltz-2, allowing us to determine the model’s reliability in high-confidence lead identification and its potential as a high-throughput alternative to computationally expensive physics-based methods.

### 3.1 Global Evaluation of Structure and Binding Affinity

We first assess the generalizability of Boltz-2 across the full data sets of 16780 (3CLPro) and 21702 (TNKS2) compounds. We compared the 3D complex structures predicted by Boltz-2 with traditional docking poses. Structural similarity was quantified using the root-mean-square deviation (RMSD). The affinity scores from Boltz-2 were compared with the ESMACS binding free energies (Δ*G*).

#### 3.1.1 Structural Comparison

To evaluate Boltz-2’s structural predictions and their reliability, we first examine the predicted protein conformations and ligand binding poses, comparing them with X-ray structures and conventional docking results. We then illustrate representative binding sites to highlight system-specific variations and potential alternative binding modes. Finally, we assess the internal consistency of Boltz-2 predictions using the confidence score, which provides a quantitative measure of the pose reliability across the ligands.

##### Protein Structure Comparison

We compare the protein structures predicted from Boltz-2 with the X-ray structures, and the ligand binding poses from Boltz-2 and the docking approach. The comparative distributions are presented in Figure 1. As a single X-ray structure (6W63 for 3CLPro and 4UI5 for TNKS2) is used in the docking approach, the Root-Mean-Square Deviations (RMSDs) for the protein reflect the variations of the predicted protein structures. A single protein conformation is predicted for 3CLPro from Boltz-2 prediction, which closely resembles the X-ray structure (RMSD ≈ 0.4 Å). For TNKS2, substantial variations are observed in Boltz-2 predicted protein conformations, as evidenced by multiple peaks in the RMSD distribution. While a small portion of the predicted structures assemble the X-ray structure, the dominant conformations are centered at 1.0 Å with some variations that extend RMSD to ∼1.8 Å from the X-ray structure.

**Figure 1:**
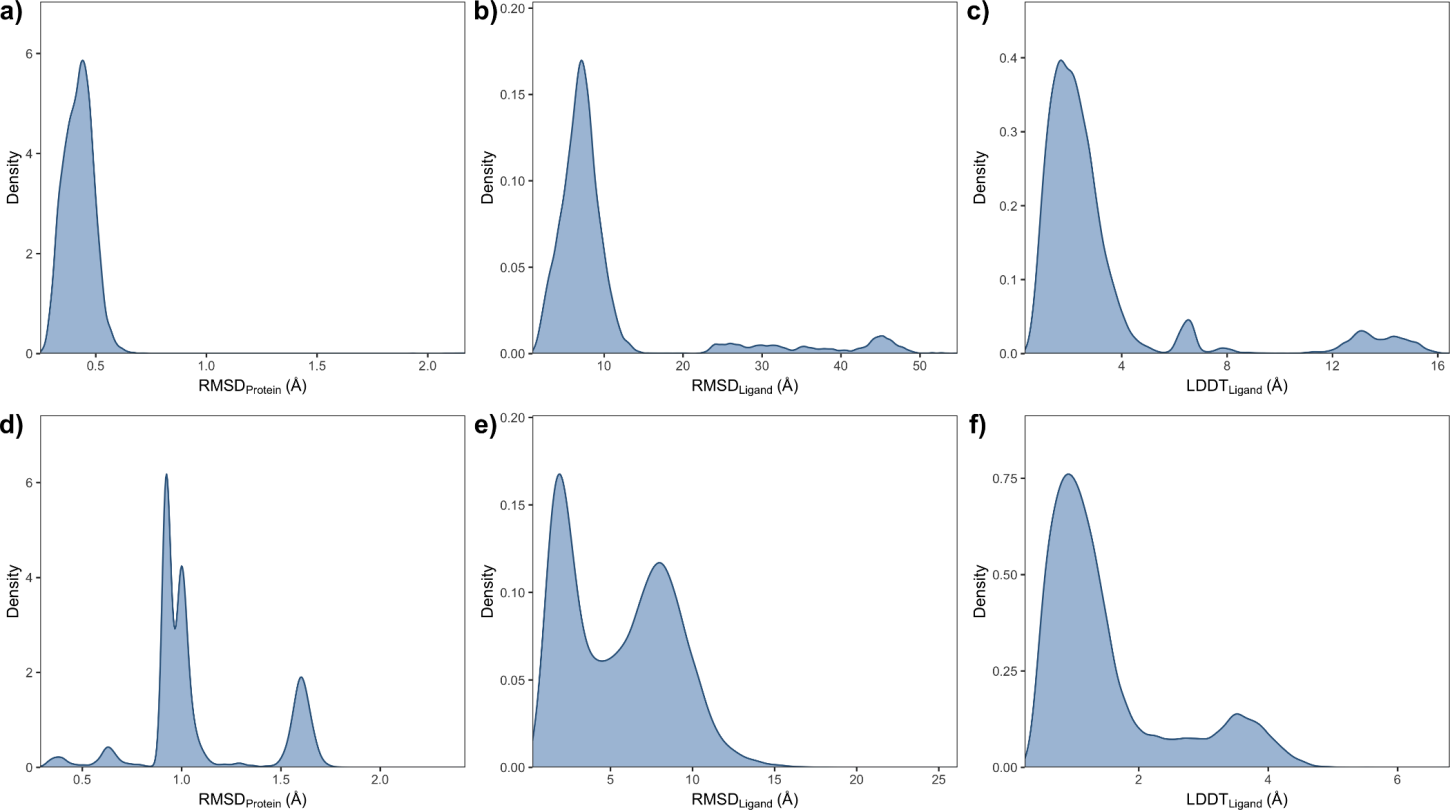
Structural comparison of Boltz-2 and docking predictions for 3CLPro (a–c) and TNKS2 (d–f). Distributions are shown for pairwise RMSDs of the protein structures (a, d) and ligand poses (b, e), alongside LDDT scores (c, f) evaluating the preservation of the local atomic environment and binding site interactions.

The RMSD values for the ligands show differences in their positions when complexed with the protein. As the binding site is explicitly defined in the docking approach, the ligands will always be in the binding site as defined in the X-ray structure. The predicted locations from Boltz-2 for some ligands, however, may not be in or near the binding site as defined in the X-ray structure (see Figure 2), resulting in large RMSDs. It should be noted that, when spatial constraints were applied, the probability of compounds being found in the catalytic pocket region can be significantly increased.^49^

**Figure 2:**
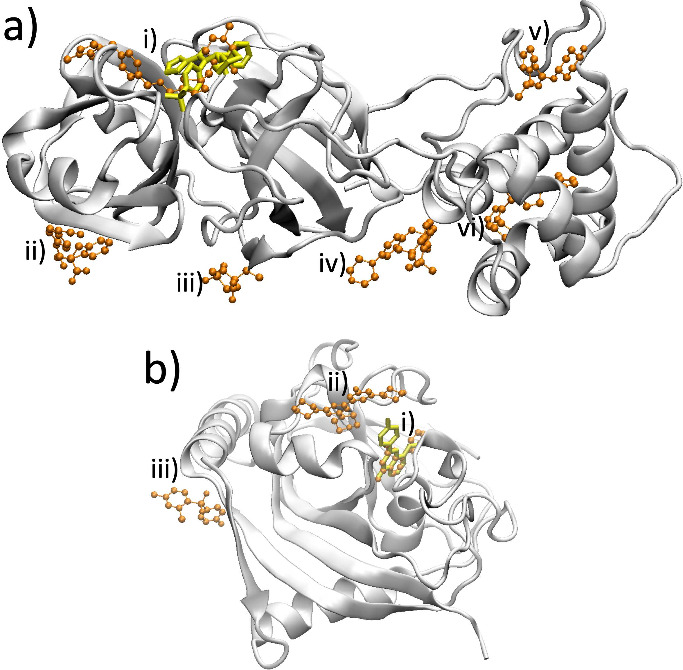
Representative binding sites predicted by Boltz-2 cofolding for (a) 3CLPro and (b) TNKS2. The protein (cartoon) and a bound compound (yellow sticks) are from PDB 6W63 and 4UI5, respectively. Five binding sites (i–vi) are predicted for 3CLPro, with LDDT metric values of 1.8, 6.4, 7.8, 13.0, 13.2, and 14.3 Å (Figure 1c), respectively. For TNKS2, all but one compounds bind at or near the X-ray identified binding site (i, ii), with the exception at a different site (iii), with LDDT values (Figure 1f) of 0.8, 3.6, and 6.9 Å, respectively.

##### Ligand Binding Pose Comparison

The analysis of ligand binding poses reveals significant system-dependent variability. For 3CLPro, the RMSD values of the ligand span a wide range, with a primary peak below 10 Å and a plateau between 22 Å and 50 Å. In contrast, TNKS2 ligands show a dual-mode distribution, with both peaks residing below 10 Å, indicating a higher accuracy of Boltz-2 predicted structures for TNKS2 than for 3CLPro when compared to docking-derived poses. The structural accuracy of predicted protein–ligand complexes is further assessed by the local distance difference test (LDDT). Unlike global RMSD metrics, LDDT is superposition-free which makes it more robust when evaluating local binding site details. The LDDT metrics mirror the shape of the ligand RMSD distributions but with metric values roughly third. LDDT is less affected by the global shifts of the ligands, and will give a high score (small LDDT value) if the ligand preserves its internal geometry and its interactions with the binding pocket, whereas ligand RMSD might be penalize heavily (large ligand RMSDs). For TNKS2, most of the LDDT metric values fall below 5 Å (see Figure 1f), and the second peak is significantly reduced from that in the ligand RMSDs (Figure 1e), indicating general agreement on the approximate location of the binding site. For 3CLPro, about 11.3% of the ligands demonstrate LDDT metrics larger than 6 Å (Figure 1c), indicating that the cofolding approach of Boltz-2 predicts binding sites that diverge significantly from the experimentally determined native site (Figure 2).

The superior pose agreement for TNKS2 is likely attributable to its narrower and more well-defined binding pocket compared with the more flexible and ambiguous binding site of 3CLPro. The observed discrepancies, particularly the second peak for TNKS2 and the wide plateau for 3CLPro in the ligand RMSD plots, arise from distinct phenomena for each protein. For 3CLPro, the similar shape of the ligand RMSD and LDDT metric distributions suggests that the peaks and the plateaus with high values correspond to ligands predicted to bind in entirely different sites. For TNKS2, the majority of the compounds bind within or adjacent to the site identified in the X-ray structure, while one exception interacts with an alternative site (Figure 2b). The significant reduction in the second peak amplitude from the protein-centric proximity profiles (LDDT in Figure 1f) indicates that some high values of ligand RMSDs are dominated by incorrect orientations, such as rotated or flipped poses, within the correct binding pocket. This distinction underscores a critical limitation in Boltz-2’s ability to resolve protein–ligand interactions precisely as the model successfully identifies the binding site but fails to accurately orient the ligand within the local topology.

##### Reliability Estimation in the Binding Pose from Boltz-2

To further characterize the reliability of these predicted ligand poses, we next examine Boltz-2’s internal confidence scores, which quantify the model’s self-assessed consistency across the ligands. The confidence score is a normalized value between 0 and 1, with higher scores indicating greater consistency of the predicted interaction according to the model’s learned representations. Importantly, this score does not correspond to experimental binding affinity but serves as a relative metric for filtering and prioritizing the algorithm’s own predictions.

To characterize predictive uncertainty, we analyzed the distribution of confidence scores and quantified the proportion of ligands exceeding a series of increasingly stringent thresholds. For each system, we calculated distribution statistics and the cumulative proportion of ligands with confidence scores above specified thresholds, with results shown in Figure 3. We observed that Boltz-2 confidence scores are strongly compressed for both systems. All ligands scored above 0.8, indicating that low-confidence predictions are essentially absent in this data set and that thresholds below 0.9 provide little discriminatory power. The distributions are highly concentrated around similar means (0.946 for 3CLPro and 0.944 for TNKS2), suggesting that Boltz-2 assigns a uniformly high internal confidence across large ligand libraries. In other words, as with all ML/AI methods, they make overconfident predictions.

**Figure 3:**
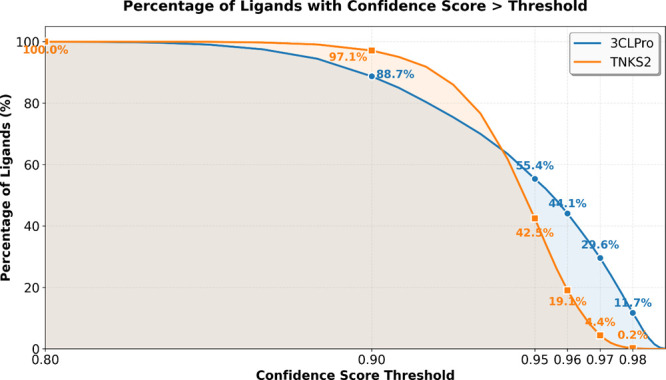
Distribution of confidence scores from the Boltz-2 predictions of both 3CLPro and TNKS2 systems. Percentages are calculated relative to the total number of ligands in each protein system.

Despite this compression, an additional structure emerges at more stringent thresholds. For 3CLPro, 55.35% of ligands exceed a confidence score of 0.95, decreasing to 44.08% above 0.96 and 29.61% above 0.97. In contrast, TNKS2 exhibits a markedly sharper drop, with only 42.46%, 19.07%, and 4.38% of ligands remaining above the respective thresholds. Thus, although TNKS2 contains a larger ligand set overall, Boltz-2 assigns high-confidence predictions to a substantially smaller fraction of compounds once strict filtering is applied.

These results reveal target-dependent behavior in Boltz-2’s internal uncertainty estimates. Although the confidence score is compressed at coarse levels, subdivision within the high-confidence regime (≥0.95) exposes meaningful separation between ligands and between targets.

Overall, our analysis reveals notable divergences between the structures predicted by Boltz-2 and docking methods (see Figure 1). This is particularly evident for 3CLPro, where a non-negligible percentage of ligands are predicted to bind at a site different to the one observed from X-ray crystallography. We cannot exclude the possibility that certain compounds, though screened or designed for the primary binding site, may occupy alternative binding sites. The more favorable binding energies observed for docked poses in our focused study (see section 3.2.2) suggest that docking-derived structures may be more reliable. Ultimately, such AI-generated structures must be rigorously validated through experimental structural determination.^6^ Indeed, a previous report has identified cases where Boltz-2 predicted PI3K-α inhibitors to bind in regions entirely distal to their confirmed orthosteric site,^38^ further underscoring the inherent risks of structural misidentification in cofolding models. This indicates yet again that caution is warranted when interpreting structures from AI-based methods. In parallel, the Boltz-2 confidence scores provide an internal measure of reliability across ligands. For both 3CLPro and TNKS2, the scores are generally high: all ligands exceed 0.80, and approximately 90% surpass 0.90. However, unless thresholds are set stringently above 0.95, the confidence score shows limited discriminatory power within these data sets. This highlights the range within which the metric is informative for assessing prediction reliability for the two systems.

#### 3.1.2 Boltz-2 Predicted Binding Affinities

While models such as AlphaFold 3 focus primarily on structural coordinates, Boltz-2 stands out through its claimed ability to predict binding affinities with precisions that approach rigorous physics-based methods. Recent benchmarks^38^ suggest it excels at qualitative ranking, effectively differentiating between potent and weak analogues within a chemical series during lead optimization. Here, we evaluate these capabilities by assessing the linear relationships and ranking fidelity of Boltz-2 relative to physics-based binding free energy calculations, utilizing Pearson correlation (*r*) and Spearman rank correlation (ρ), respectively.

Our previous studies across diverse molecular systems have demonstrated that ESMACS reliably ranks binding affinities, despite a characteristic overestimation of absolute free energies. While no universal threshold exists due to the system-dependent nature of the overestimation, compounds with ESMACS values exceeding −20 kcal/mol are generally considered nonbinders or extremely weak hits.^50^ In contrast, Boltz-2 exhibits a “regression to the centre” effect, where predictions are concentrated within a remarkably narrow range, with the vast majority of affinities falling between −5 and −8 kcal/mol. The model maintains these relatively strong predicted affinities even for probable nonbinders, indicating an evident lack of sensitivity in distinguishing true hits from decoys (nonbinding molecules sharing similar 1D or 2D characteristics with the hits) based solely on the predicted binding affinities. Consequently, both Δ*G*_Boltz_ and Δ*G*_ESMACS_ are subject to inherent, disparate errors and exhibit significant numerical misalignment, making their absolute values not directly comparable. To account for this, we employed standardized major axis (SMA) regression for linear fitting. Unlike ordinary least-squares (OLS), which assumes error only in the dependent variable and often suffers from slope attenuation (regression dilution), SMA minimizes the area of right triangles formed by both vertical and horizontal residuals. This ensures the fitted line remains centrally aligned within the data cloud, providing a more symmetric representation of the relationship between two error-prone variables.

##### Correlation of Predicted Binding Affinity between ESMACS and Boltz-2

While Boltz-2 yields a moderate correlation for TNKS2 (Pearson *r* = 0.45 and Spearman ρ = 0.46, see Figure 4), it exhibits more limited accuracy for 3CLPro (*r* = 0.24 and ρ = 0.25). This discrepancy can be attributed to distinct binding site architectures: while 3CLPro possesses a large and branched binding site that introduces conformational and energetic variability in ligand bindings, TNKS2 features a narrow and well-defined binding pocket that enables more reliable pose and energy determination. The structural differences in the binding sites may also account for the more accurate prediction of binding probabilities for TNKS2. As shown in Figure 4b, TNKS2 exhibits a visually clearer clustering of high-probability points within the high-affinity (more negative Δ*G*) region compared to 3CLPro. However, only weak correlations are observed between the Boltz-2 predicted binding affinities and binding probabilities, with Pearson correlation coefficients of 0.21 and 0.25 for 3CLPro and TNKS2, respectively. Almost all compounds are predicted by Boltz-2 to have binding affinities below −4 kcal/mol, even though many are assigned very low binding probabilities. Despite their different roles as classification and regression heads, the binding affinity and binding probability derive from the same structural embeddings and should manifest a consistent correlation between their outputs. The weak correlation observed in the current study indicates an inconsistency in the model’s multiobjective architecture.

**Figure 4:**
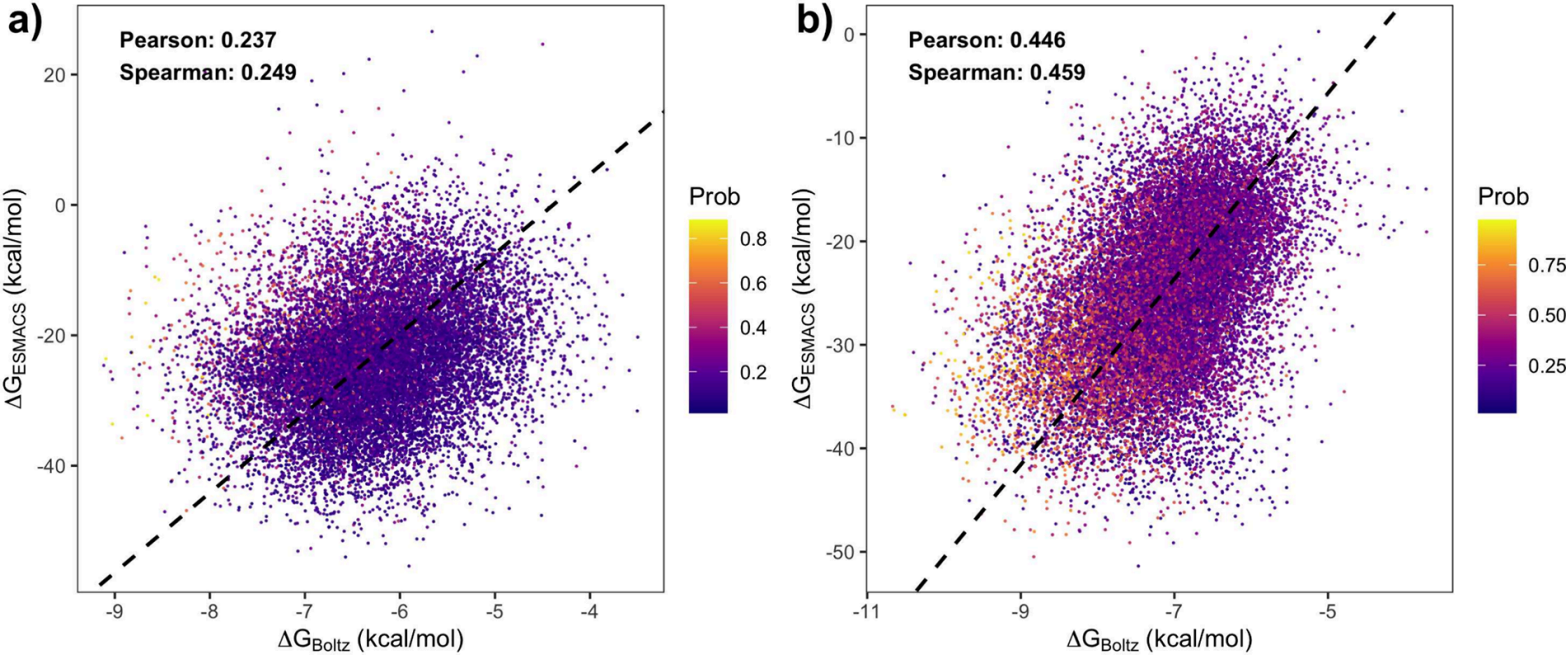
Correlation between binding free energies predicted by Boltz-2 (Δ*G*_Boltz_) and calculated via ESMACS (Δ*G*_ESMACS_) for (a) 3CLPro and (b) TNKS2. Dashed lines indicate standardized major axis (SMA) regressions. Data points are colored by Boltz-2 predicted binding probabilities.

##### Reproducibility of Boltz-2

To evaluate the internal consistency, Boltz-2 predictions were compared between independent inference runs for each protein system. We used the same default pretrained model weights for these runs, and hence the aleatoric uncertainties from random seeds during its trainings were not accounted for in our repeated runs.

For this study, 793 ligands for 3CLPro and 799 ligands for TNKS2 were independently re-evaluated with Boltz-2. Reproducibility was quantified using Pearson and Spearman correlation between the two runs. As shown in Figure 5, both 3CLPro and TNKS2 predictions showed high reproducibility, with Pearson *r* of 0.913 and 0.962, and Spearman ρ of 0.897 and 0.954, respectively, indicating that Boltz-2 predictions are generally stable. Meanwhile, a slight system-dependent difference was observed, with TNKS2 showing better alignment between runs than 3CLPro. Nevertheless, despite the strong overall correlation, absolute differences in predicted binding affinities between runs can reach ∼1.5 kcal/mol. Such deviations are significant for compound ranking, potentially shifting the priority of lead candidates within the model’s relatively narrow prediction range (approximately −4 to −9 kcal/mol for the majority of ligands).

**Figure 5:**
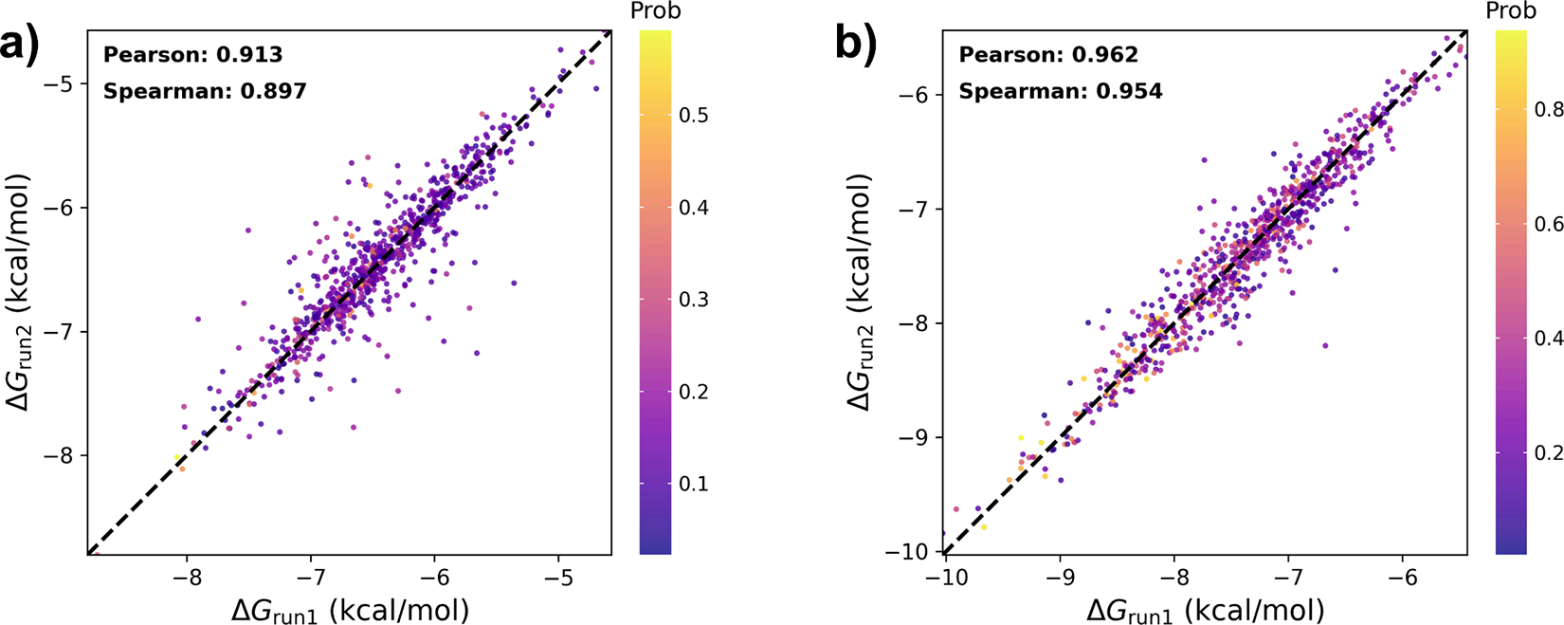
Binding affinity consistency between two independent runs of Boltz-2 for (a) 3CLPro and (b) TNKS2.

### 3.2 Precision Analysis of Top-100 Boltz-2 Predictions

To evaluate the utility of Boltz-2 as a primary filter for virtual screening, we analyzed the top 100 compounds ranked according to the predicted affinity of Boltz-2 for each target. It should be noted that the overlap between the top 100 compounds identified by Boltz-2 and ESMACS was minimal, with only 2 and 1 compound(s) in common for 3CLPro and TNKS2, respectively. Statistical analysis reveals that for data sets of these sizes (*N* = 16780 and 21702), the probability of finding at least one common compound by random chance is 45.0% and 36.9%, respectively. This suggests that the two scoring methodologies explore fundamentally different regions of the chemical landscape.

#### 3.2.1 Binding Poses from Boltz-2

During the evaluation of Boltz-2-generated poses, a systematic discrepancy was observed between the protonation and saturation states of the predicted ligand structures and their canonical biochemical forms. The Boltz-2 predicted structures contain only heavy atoms (C, N, O, S, etc.). Explicit hydrogen atoms were added with OpenEye tools to the structures. For a subset of these top 100 compounds, 28 for 3CLPro and 30 for TNKS2, the atomic models exhibited divergent hydrogen counts and corresponding indices of hydrogen deficiency (IHDs) relative to their defining SMILES strings.

This deviation manifests primarily in two distinct patterns: (1) for ring systems, predicted structures frequently show increased unsaturation; and (2) for aliphatic chains, Boltz-2 predicted models display a bias toward increased saturation. Aromatic or alicyclic rings in the SMILES are often predicted in a more unsaturated, often fully conjugated state. For example, a pyrrolidinyl moiety (saturated, IHD = 1) is predicted as a pyrrolyl group (aromatic, IHD = 3) and a piperidinyl moiety (saturated, IHD = 1) as a dihydropyridinyl group (IHD = 3) (see Figure 6a,c). In contrast, unsaturated carbons in aliphatic chains (e.g., vinyl group with IHD = 1, Figure 6d) are sometimes predicted as saturated *sp* ^3^-hybridized ones (e.g., ethyl group with IHD = 0, Figure 6b), effectively adding hydrogen atoms not present in the original specification.

**Figure 6:**
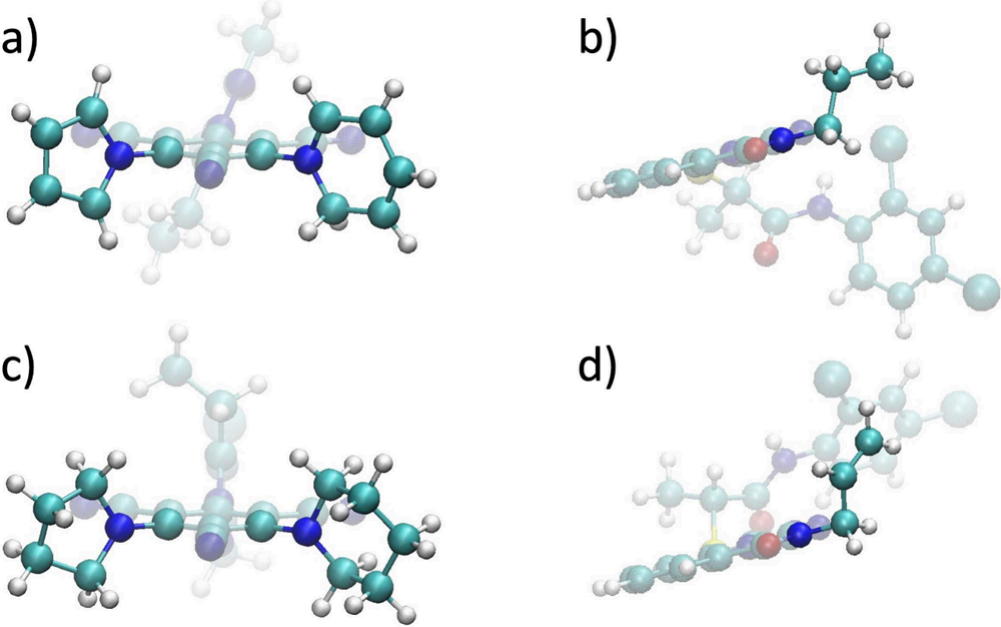
Comparison of saturation state deviations. Boltz-2 saturation is derived from hydrogen placement on predicted heavy-atom coordinates. Representative deviations of the saturation states from Boltz-2 predictions are shown for rings (a) and aliphatic chains (b), alongside their SMILES string counterparts (c, d).

We hypothesize that these opposing biases stem from Boltz-2’s training on structural data sets and their implicit energy landscape. For rings, the model may favor planar, conjugated geometries that represent deep local minima in vacuum or implicit-solvent potentials. For chains, the model appears to prioritize sterically relaxed, tetrahedral geometries, potentially smoothing out conjugated patterns that are stabilized specifically by protein–ligand interactions or explicit electronic effects. Passaro et al.^29^ explicitly list some significant physical issues with the poses Boltz-2 generates, including “slightly incorrect bond lengths and angles, incorrect stereochemistry at chiral centers and stereobonds and aromatic rings predicted to be nonplanar”. This is precisely the reason for the saturation state deviations we observe here.

These systematic alterations in ligand bonding topology have direct consequences for binding affinity predictions. Changes in hybridization and saturation often substantially affect molecular shape, steric complementarity with the binding pocket, electrostatic potential, hydrogen-bonding capacity, and local flexibility and entropic contributions to binding, which are all critical determinants of binding free energy. To isolate the source of prediction error, we performed two parallel sets of affinity calculations: (1) using the Boltz-2 predicted structures with hydrogens added according to the model’s implicit bonding topology; and (2) using canonical and reprotonated structures, where the heavy-atom coordinates from Boltz-2 were retained, but all bonding and hydrogenation states were corrected to match the definitive SMILES representation. This dual-pathway approach allows us to distinguish between errors arising solely from 3D pose geometry and those compounded by an incorrect chemical identity. The comparative analysis of these two affinity sets, presented in section 3.2.2, quantifies the impact of saturation state artifacts on the overall performance of the Boltz-2 pipeline.

#### 3.2.2 Binding Free Energies

Although there are weak to moderate overall correlations for the entire data sets for 3CLPro and TNKS2 (Figure 4), no correlation is observed between the initial Boltz-2 predictions and the ESMACS calculations for the top 100 compounds selected (Figure 7a,b,e,f), no matter what saturation (Figure 6) and/or protonation states assigned for the compounds. The Pearson and Spearman correlation coefficients are close to zero. It is not uncommon for correlations to “break down” when zooming in on the top-performing candidates. The selection of top 100 compounds significantly reduces the variance of the Boltz-2 predicted binding affinities (within 1.0 kcal/mol of each other), which is likely to be smaller than the model’s inherent uncertainty, making the correlation mathematically collapses. Furthermore, the reported insensitivity of Boltz-2 affinities to structural accuracy^34^ explains the lack of correlation with our ESMACS calculations which responds to the specific physical environment of the binding site. Similar to other cofolding approaches that do not explicitly account for physics-based interactions during structure prediction,^9^ Boltz-2 appears to derive binding affinities from features largely independent of the final ligand pose.^34^ Therefore, the binding affinities predicted from Boltz-2 are not a reliable quantitative predictor of the more rigorous, physics-based ESMACS binding free energies for this specific set of 100 compounds. This indicates that Boltz-2 may be effective for classification (identifying binders), but does not provide accurate binding free energies compared to simulation-based approaches.

**Figure 7:**
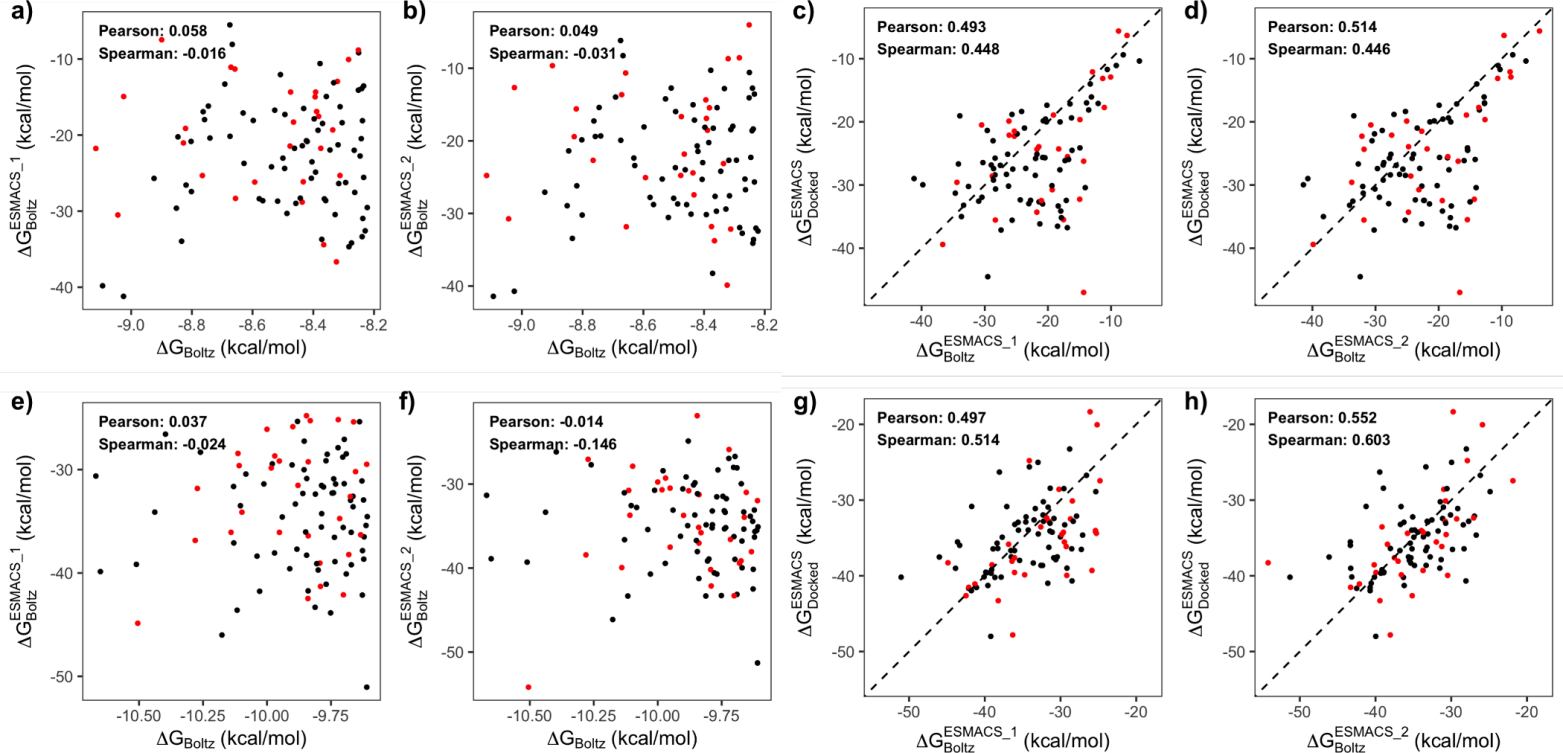
Comparison of binding free energies from Boltz-2 prediction (Δ*G*_Boltz_) and ESMACS simulations. Three sets of ESMACS simulations are performed, denoted as Δ*G*_Boltz_^ESMACS_1^, Δ*G*_Boltz_^ESMACS_2^ and Δ*G*_Docked_^ESMACS^, respectively, initiated from structures derived directly from Boltz-2 predicted structures, derived from SMILES strings with positions of heavy atoms from Boltz-2 predictions, and from docked approach. Some compounds have different saturation or protonation states for Δ*G*_Boltz_^ESMACS_1^ and Δ*G*_Boltz_^ESMACS_2^ calculations; they are shown as red dots.

There is a moderate positive correlation between two different ESMACS calculations that use different initial structural inputs (Boltz-generated vs docked structures), with correlation coefficients ranging from approximately 0.45 to 0.60 (Figure 7c,d,g,h). Therefore, the ESMACS calculations are relatively consistent, showing moderate agreement between runs initiated with different but likely viable binding poses. The moderate correlation suggests general agreement in binding strength, although specific values may vary slightly. Compounds with different saturation and/or protonation states do not appear systematically as major outliers, although some deviation is present (Figure 7c,d,g,h). The corrected saturation or protonation states improve the correlations for TNKS2, although their impact is less significant for 3CLPro.

While the data points cluster reasonably well around the diagonal dashed line (line of identity, where x = y), there are more data points below the line: 66 out of 100 compounds for 3CLPro (Figure 7c) and 60 out of 100 for TNKS2 (Figure 7g). The correction for saturation and protonation scarcely changes the trend, with 67 and 59 data points below the line for 3CLPro and TNKS2 (Figure 7d,h), respectively. It is important to note that these compounds were originally identified through an iterative active learning protocol for optimal molecular design,^21^ which coupled the generative AI framework REINVENT with the physics-based ESMACS approach. Both REINVENT and ESMACS serve as core components within the IMPECCABLE workflow, and the current results indicate that for these expertly designed molecules, the docked structures consistently yield more favorable binding free energies than those predicted by Boltz-2.

### 3.3 Experimental Validation against BindingDB

To address the critical requirement of validating our computational predictions against direct experimental evidence, we evaluated both Boltz-2 and ESMACS using experimental binding data retrieved from the BindingDB database for the two protein targets in this study: TNKS2 and 3CLPro. However, due to a severe scarcity of available experimental binding data for 3CLPro, our evaluation in this section focuses primarily on the TNKS2 system, which features a robust data set of over 1000 compounds in the database.

For TNKS2, Boltz-2 demonstrated a strong correlation with experimental measurements (*r* = 0.77 and ρ = 0.78), whereas the physics-based ESMACS protocol yielded a significantly lower correlation (*r* = 0.23 and ρ = 0.23) (see Figure 8). For 3CLPro, Boltz-2 maintained a weak correlation, while ESMACS displayed no statistical correlation with the experimental values. The observed performance differences point directly to the fundamental difference between data-driven machine learning models and physics-based free energy protocols. Deep learning models are inherently optimized to average out structural and experimental noise over a large number of compounds. While ESMACS has been demonstrated to be highly reliable for the ranking of binding affinities for many molecular systems,^17–19,43,44^ the primary driver behind the apparent poor correlation of ESMACS here is likely to be the severe interlaboratory experimental heterogeneity characteristic of public data repositories. The experimental data in BindingDB represents an assembly of independent studies conducted over many years, subsuming numerous variables including assay conditions, experimental setups, measurement techniques, enzyme and substrate variations, and so on.

**Figure 8:**
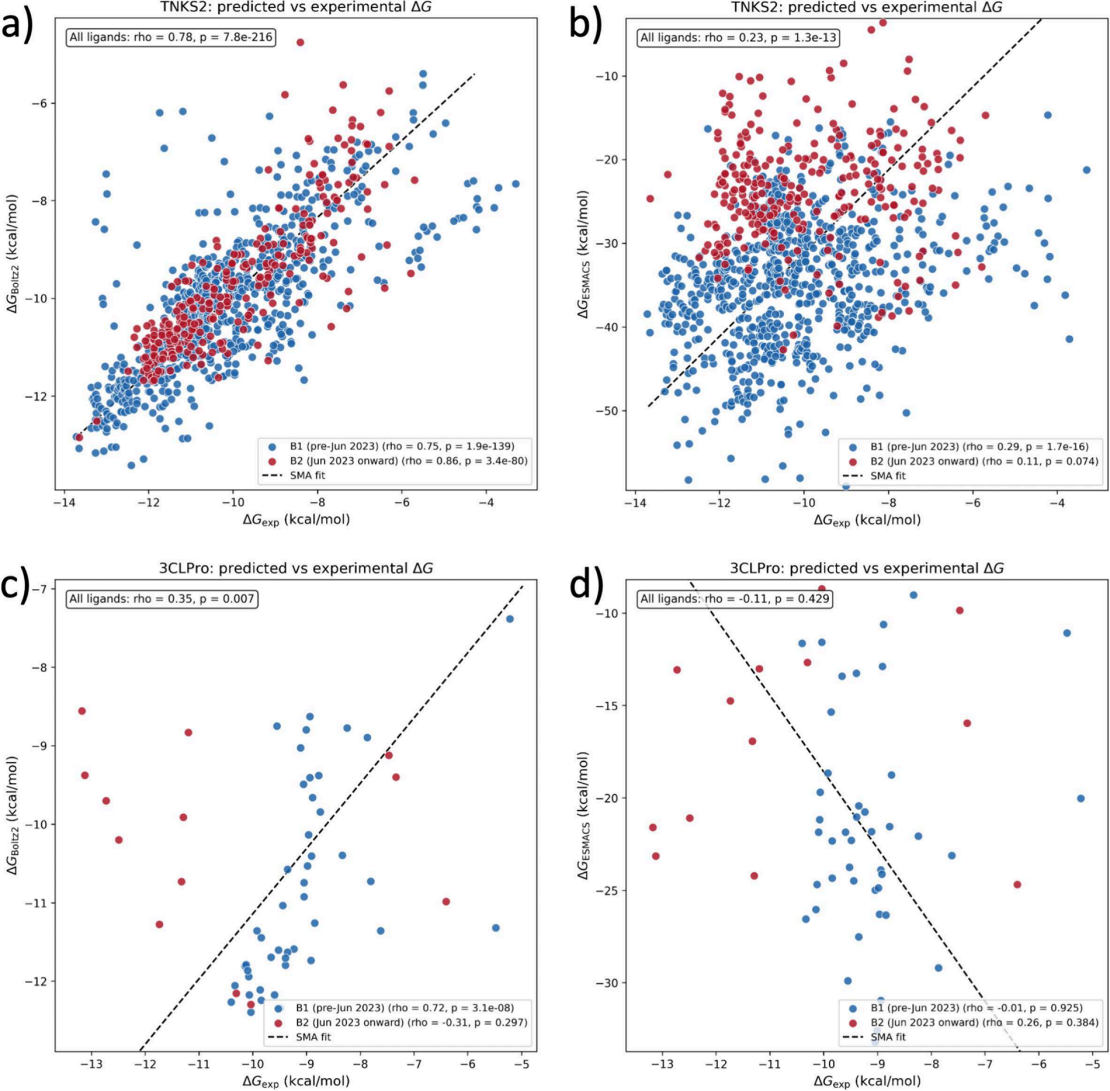
Correlations of binding free energies obtained from Boltz-2 (a, c) and ESMACS (b, d) with experimental measurements of compounds from BindingDB for TNKS2 (a, b) and 3CLPro (c, d). The data is split into B1 (pre-June 2023, blue) and B2 (June 2023 onward, red) to track performance stability over time.

Substantial data heterogeneity and intrinsic uncertainty therefore exist within these public repositories. The severity of this noise is vividly demonstrated within the evaluated TNKS2 data set itself. Our analysis reveals multiple chemical compounds for which independent laboratories reported vastly conflicting binding metrics. In the most severe instances, the discrepancy in binding free energies reaches up to 5.1 kcal/mol for the exact same ligand binding to TNKS2, implying a shift in IC_50_ or *K*_*i*_ of roughly 3.7 to 4 orders of magnitude. Literature benchmarks confirm this vulnerability; meta-analyses of broad public data sets demonstrate that even minimal curation leaves over 27% of overlapping IC_50_ data points differing by more than 1.0 log unit (1.4 kcal/mol), yielding poor cross-experimental correlations (τ ≈ 0.51).^51^

To further quantify the baseline experimental variance within the data set, we analyzed a subset of 271 compounds for the TNKS2 data set that possessed multiple experimental measurements. We implemented a bootstrapping approach over 10000 iterations, where two distinct vectors were generated by randomly sampling a single experimental value for each compound per iteration. The mean Pearson correlation coefficient between these randomly sampled experimental pairs was *r* = 0.56 with a substantial standard deviation of σ = 0.25. This remarkably low internal correlation demonstrates that a single compound can exhibit highly divergent reported affinities across different literature sources or assay conditions within the same repository. Furthermore, high-volume experimental data often follow non-Gaussian distributions, rendering standard averaging methods insufficient for resolving such noise.^52^ The pronounced experimental noise imposes a strict mathematical ceiling on how well any computational model can fit the raw data. The apparent high correlation (*r* = 0.77) between the Boltz-2 predictions and the experimental measurements indicates that the model may be fitting underlying experimental noise rather than true biological signal.

The temporal split for TNKS2 — divided into B1 (pre-June 2023) and B2 (June 2023 onward) — reveals a similar correlation between the Boltz-2 predictions and the experimental measurements (Figure 8). While the superior correlation of Boltz-2 after its training cutoff date is promising, it must be interpreted with caution regarding true generalization. The compounds added to BindingDB after the model’s training threshold still share a high degree of structural similarity and soft overlap in chemical space with the historical training data. As illustrated by the t-SNE projections in Figure 9, the vast chemical space represented by the blue dots consists of novel compounds generated by the generative AI framework, REINVENT. In sharp contrast, the experimental compounds from BindingDB occupy only a highly localized, narrow and structurally constrained region of this space. Crucially, the B2 compounds do not venture into unexplored chemical territory; instead, they cluster tightly within the exact same narrow islands already defined by the historical B1 data. The high correlation observed for Boltz-2 is likely to stem from the model’s memory of closely related chemical scaffolds, rather than a rigorous prediction of thermodynamic binding affinities. Consequently, these findings highlight that large uncertainties are inherently embedded in public repositories like BindingDB. Researchers must exercise extreme caution when using such data sets to evaluate, rank or validate computational binding affinity prediction workflows, as the experimental “ground truth” itself possesses significant variance.

**Figure 9:**
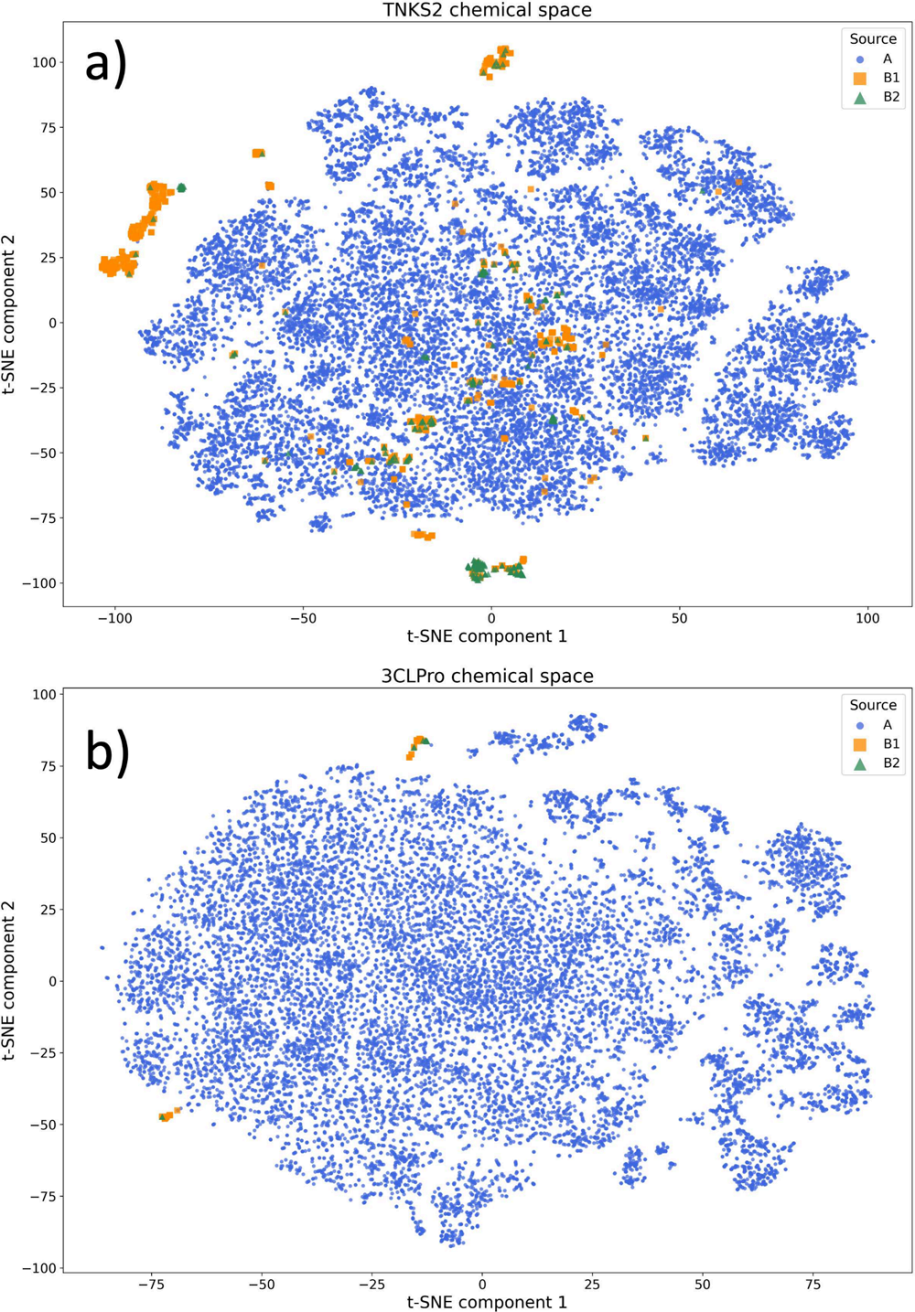
Chemical space visualization via t-SNE. Projection of the first two t-SNE components based on Tanimoto fingerprints for compounds from successive GAL iterations (A) and BindingDB (B1 and B2) for TNKS2 (a) and 3CLPro (b). While the GAL-derived compounds occupy a significantly broader chemical space, the BindingDB reference compounds are confined to a much narrower region. Furthermore, BindingDB compounds from before (B1) and after (B2) the Boltz-2 training cutoff date exhibit high structural overlap.

Because ESMACS operates on foundational physics principles, it typically models a singular, localized but dynamical biophysical state. It cannot simultaneously correlate with potentially conflicting experimental profiles that span an up to 5.1 kcal/mol margin of error. The fact that ESMACS achieves a correlation of 0.23 for TNKS2 across such corrupted input values proves its operational sensitivity rather than a failure of its physics engine. When applied to highly controlled, single-source intralaboratory workflows — where assay noise is tightly constrained — ensemble methods like ESMACS routinely yield high predictive precision.^17^ Consequently, ESMACS remains a highly reliable approach for evaluation of novel scaffolds where no prior data exists. By establishing this rigorous physical baseline, we have verified that our evaluation data set is an ideal stress-test for Boltz-2. It allows us to clearly differentiate between true thermodynamic prediction and the localized scaffold memorization inherent to machine learning models.

## 4 Conclusions

We have conducted a rigorous evaluation of the Boltz-2 foundation model’s capability to predict protein–ligand structures and binding affinities across two large-scale data sets, 3CLPro and TNKS2. By benchmarking the model’s performance against traditional docking and the physics-based ESMACS protocol, we have identified several critical limitations within the current state of AI-driven structural biology, which are summarized here.

Our structural analysis reveales significant global RMSD variations in both protein and ligand conformations. These findings indicate that, while Boltz-2 captures a broader conformational ensemble than traditional static docking, it does not consistently identify a single, dominant “native” binding site across diverse chemical libraries. It should be noted that Boltz-2 can apply geometric constraints like pocket definitions to steer compounds to a predefined binding site. It can also employ Boltz-steering,^29^ a physics-based potential, to make predictions away from physically implausible states. By enforcing physical realism, structure and pose validity can be improved, albeit at the cost of additional checks that increase memory overhead and inference time.

Regarding binding energetics, Boltz-2 demonstrates only weak to moderate correlations with physics-based free energy values in the global evaluation. More strikingly, the focused analysis of the top 100 compounds revealed a complete lack of correlation with fine-grained ESMACS results. This indicates that, while Boltz-2 is efficient at broad-spectrum screening, it lacks the resolution required for the hit-to-lead phase, where ranking structural analogues to establish clear structure–activity relationship is critical. The observation of hydrocarbon “saturation” issues for certain ligands further indicates that the model is not able to accurately predict the binding poses for some ligands, and consequently their binding affinities which rely on the structures it predicts.

The root of these limitations lies in a fundamental assumption most machine learning architectures rely on: that biomolecular data behaves as a smooth, differentiable manifold. Rather than explicitly incorporating the underlying physics of biomolecular interactions, ML-based approaches rely on statistical patterns from training data sets. The assumption of differentiability often fails when navigating the vast chemical space, where structure–activity relationships (SARs) are often discontinuous. A primary manifestation of this is the “activity cliff”,^53^ where minor structural modifications lead to disproportionate changes in binding affinity. Such nonlinear phenomena are inherently difficult for current AI architectures to navigate, underscoring the necessity of integrating fundamental physics to both interpret model failures and develop more robust algorithms.^54^

In conclusion, while Boltz-2 offers an efficient tool for generating protein–ligand complexes when canonical docking approaches are unfeasible, its poor performance in affinity ranking indicates that it cannot serve as a reliable substitute for high precision, physics-based methods in late-stage drug discovery. Our results suggest a fundamental incompatibility between the model’s smooth, differentiable assumptions and the nonlinear, discontinuous nature of the physical energy landscape. Rather than learning the underlying physics of molecular recognition, the model appears to rely on *ad hoc* statistical heuristics that are largely decoupled from local atomic environments. This vulnerability is exacerbated by the “deluge of spurious correlations” inherent in massive data sets. As demonstrated by Calude and Longo,^55^ the ratio of false to true correlations increases combinatorially as a result purely of the size of a data set, not its nature. One needs to find ways of identifying the true correlations to weed out all the false ones. This requires scientific understanding applied by a form of reinforcement learning. Until foundation models can move beyond these mathematical artifacts to incorporate the rigor and uncertainty quantification of physics-based interactions, they will remain insufficient for navigating the complex activity cliffs of real-world chemical space.

## Data Availability

All data sets generated in this study are deposited in the Zenodo repository (10.5281/zenodo.21041917). This collection includes Boltz-2 predicted structures, binding affinities and binding probabilities for 16780 compounds targeting 3CLPro and 21702 compounds targeting TNKS2, alongside their corresponding coarse-grained ESMACS binding free energies. The repository also contains structural coordinates — derived from both Boltz-2 and docking — and high-resolution, fine-grained ESMACS free energy calculations for the top 100 selected compounds. Additionally, data for compounds extracted from BindingDB, including their predicted binding affinities from both Boltz-2 and ESMACS calculations, are provided.
